# Inhibition of Glycolysis Alleviates Chronic Unpredictable Mild Stress Induced Neuroinflammation and Depression-like Behavior

**DOI:** 10.3390/brainsci14111098

**Published:** 2024-10-30

**Authors:** Bing Liu, Ke Dong, Xiaobing Chen, Huafeng Dong, Yun Zhao, Xue Wang, Zhaowei Sun, Fang Xie, Lingjia Qian

**Affiliations:** 1 Beijing Institute of Basic Medical Sciences, #27 Taiping Road, Haidian, Beijing 100039, China; liubingzsp@163.com (B.L.); dongke0302@163.com (K.D.); 17859518569@163.com (X.C.); feng13191908265@163.com (H.D.); 15710282409@139.com (Y.Z.); snowwang0326@foxmail.com (X.W.); sunzhw0820@163.com (Z.S.); vancoxie1@163.com (F.X.); 2 School of Medicine, South China University of Technology, Guangzhou 511442, China

**Keywords:** CUMS, neuroinflammation, microglia, glycolysis, 2-DG

## Abstract

Background: Growing evidence suggests that glucose metabolism plays a crucial role in activated immune cells, significantly contributing to the occurrence and development of neuroinflammation and depression-like behaviors. Chronic stress has been reported to induce microglia activation and disturbances in glucose metabolism in the hippocampus. Aims: This study aims to investigate how chronic stress-mediated glycolysis promotes neuroinflammation and to assess the therapeutic potential of the glycolysis inhibitor, 2-deoxy-D-glucose (2-DG), in a model of chronic stress-induced neuroinflammation and depression-like behavior. Methods: In in vitro studies, we first explored the effects of 2-DG on the inflammatory response of microglia cells. The results showed that corticosterone (Cort) induced reactive oxygen species (ROS) production, increased glycolysis, and promoted the release of inflammatory mediators. However, these effects were reversed by intervention with 2-DG. Subsequently, we examined changes in depression-like behavior and hippocampal glycolysis in mice during chronic stress. The results indicated that chronic stress led to prolonged escape latency in the Morris water maze, increased platform-crossing frequency, reduced sucrose preference index, and extended immobility time in the forced swim test, all of which are indicative of depression-like behavior in mice. Additionally, we found that the expression of the key glycolytic enzyme hexokinase 2 (HK2) was upregulated in the hippocampus of stressed mice, along with an increased release of inflammatory factors. Further in vivo experiments investigated the effects of 2-DG on glycolysis and pro-inflammatory mediator production, as well as the therapeutic effects of 2-DG on chronic stress-induced depression-like behavior in mice. The results showed that 2-DG alleviated chronic stress-induced depression-like behaviors, such as improving escape latency and platform-crossing frequency in the Morris water maze, and increasing the time spent in the center of the open field. Additionally, 2-DG intervention reduced the level of glycolysis in the hippocampus and decreased the release of pro-inflammatory mediators. Conclusions: These findings suggest that 2-DG can mitigate neuroinflammation and depressive behaviors by inhibiting glycolysis and inflammatory responses. Overall, our results highlight the potential of 2-DG as a therapeutic agent for alleviating chronic stress-induced neuroinflammation through the regulation of glycolysis.

## 1. Introduction

In contemporary society, ongoing stress has become a crucial element affecting people’s well-being. Prolonged exposure to stress can elicit a range of physiological and psychological responses, with depressive-like behaviors being among the most prevalent manifestations. Chronic stress not only adversely affects an individual’s emotional state but also modifies behavior and cognitive functions by regulating the activities of the central nervous system (CNS), endocrine system, and immune system [1,2,3]. Studies suggest a significant link between chronic stress and the development and worsening of depression, with various complex biological processes playing a role, including abnormal activation of the hypothalamic–pituitary–adrenal (HPA) axis, heightened neuroinflammation, and imbalances in neurotransmitter systems [4,5,6]. These biological mechanisms interact synergistically, rendering individuals under stress more vulnerable to depressive-like behaviors. Exploring how chronic stress leads to the emergence of depressive-like behaviors is crucial for understanding the pathophysiological mechanisms of depression and for discovering effective treatment options.

Growing evidence indicates that inflammation plays a key role in diseases linked to stress-induced damage [7]. Recent research shows that chronic stress can activate immune cells in the brain and initiate the release of inflammatory mediators [8]. Microglia, the resident immune cells in the CNS, are a primary source of neuroinflammation caused by chronic stress [9]. When faced with immune challenges, microglia become activated, undergoing significant morphological changes. This pro-inflammatory activation results in the release of large amounts of inflammatory factors, such as interleukin-6 (IL-6), interleukin-1β (IL-1β), and tumor necrosis factor-α (TNF-α), promoting the onset of neuroinflammation [10,11,12]. Consequently, regulating microglia activation and the associated neuroinflammation may represent a crucial strategy for mitigating cognitive impairment induced by chronic stress.

The phenotype and metabolic state of microglia frequently shift in response to immune activation [13,14]. Classically activated microglia switch their metabolic pathway from oxidative phosphorylation to aerobic glycolysis, a process known as the Warburg effect. Specifically, lipopolysaccharide (LPS) induces this metabolic shift toward aerobic glycolysis, leading to the release of early pro-inflammatory cytokines. In contrast, alternatively activated anti-inflammatory microglia show an increased mitochondrial basal oxygen consumption rate (OCR) [15,16]. Metabolic dysregulation is also observed in organisms under stress and can interact with inflammatory responses [17]. Our previous results indicate that chronic stress can induce glucose metabolic disorders in the hippocampus of mice while simultaneously activating the pro-inflammatory phenotype of microglia [18]. This metabolic alteration underscores the varying energy demands of microglia in different states. Furthermore, additional studies have demonstrated that the inflammatory response could induce abnormalities in glucose metabolism, resulting in hyperglycemia [19,20,21]. Given the critical roles of glucose metabolism dysregulation and inflammatory responses in stress reactions, targeting glucose metabolism may offer new insights into the treatment of neuroinflammatory diseases. In recent years, the role of 2-DG in chronic stress has attracted growing interest from researchers. Studies on 2-DG in the context of chronic stress mainly focus on its dual impact on both the nervous and metabolic systems.

Metabolism, as a fundamental process underlying biological phenomena, provides energy and serves as the foundation for life activities. 2-DG, a glucose analog, is phosphorylated by hexokinase (HK) to 2-DG-phosphate but cannot be further metabolized, which disrupts cellular energy supply and function [22]. In recent years, the role of 2-DG in chronic stress has attracted growing interest from researchers. Studies on 2-DG in the context of chronic stress mainly focus on its dual impact on both the nervous and metabolic systems [22,23,24]. Preliminary studies suggest that 2-DG may mitigate some negative effects of the stress response by disrupting glucose metabolism [23,25]. For instance, studies have demonstrated that 2-DG can inhibit the overactivation of the HPA axis, decrease the secretion of stress hormones, and alleviate behavioral and physiological abnormalities associated with chronic stress [26,27]. Furthermore, 2-DG contributes to ameliorate stroke and Alzheimer’s disease by regulating energy metabolism and reducing inflammatory responses [28]. In this context, our main objective is to clarify the role of glycolytic abnormalities in neuroinflammatory responses triggered by chronic stress. Our study found that chronic stress can elevate glycolysis in the hippocampus of mice, which is positively correlated with microglia inflammatory polarization and cognitive impairment. Furthermore, administering 2-DG to chronically stressed animals not only inhibited hippocampal glycolysis and neuroinflammation but also alleviated depressive-like behavior in mice. These results suggest a potential new strategy for treating stress-related depressive-like behaviors.

## 2. Materials and Methods

### 2.1. Animals Experiment

Male wild-type C57BL/6 mice (6–7 weeks old) were obtained from HuaFuKang Company (Beijing, China). All procedures were carried out in accordance with the National Institutes of Health Guide for the Care and Use of Laboratory Animals (NIH Publication No. 8023) and were approved by the Institutional Animal Care and Use Committee of the AMS. The mice were housed under controlled conditions with free access to rodent food and tap water, except during stress interventions and behavioral experiments. After completing the behavioral tests, the mice were anesthetized intraperitoneally with tribromoethanol (Meilunbio, MA0478) at a dose of 30 µL/g body weight, and brain tissues were collected for further analysis. One week after acclimatization, mice (*n* = 45) were randomly divided into three treatment groups: control group, CUMS, and 2-DG. Then, Elisa (*n* = 10), QPCR (*n* = 6), WB (*n* = 3), and IF (*n* = 3) were performed.

### 2.2. Chronic Unpredictable Mild Stress (CUMS)

After one week of acclimation to the housing conditions, the mice underwent 8 weeks CUMS modeling, following established protocols [29,30]. In short, the mice were exposed to two random stressors daily, including 24 h of fasting, 24 h of water deprivation, 12 h of wet bedding, 24 h of cage tilting at a 30° angle, 6 h of body restraint, 2 min of forced swimming in 4 °C water, 30 min of shaking at 120 rpm, and overnight illumination. The control group was kept under standard conditions.

### 2.3. Morris Water Maze (MWM)

The Morris water maze test was used to evaluate spatial learning and memory in mice, following a modified version of the previous experimental protocol [31]. The experiment took place in a circular water maze with a diameter of 1.2 m, with the water temperature maintained at 19–22 °C, in a quiet lab environment free from external disturbances. Mice underwent daily training sessions lasting 3 min each, once a day for 5 consecutive days. During training, each mouse had 180 s to swim freely and locate the platform. If a mouse failed to find the platform, it was guided to it and allowed to stay there for 30 s. The time taken to find the hidden platform was recorded as escape latency. In the testing phase, the platform was removed, and the mouse was placed in the water facing the wall opposite the quadrant where the platform had been. The time taken for the mouse to first reach the platform location and the number of times it crossed the platform area within 3 min were recorded. Mouse movements were tracked using the Noldus EthoVision XT 16 system from Noldus Information Technology BV, Wageningen, The Netherlands.

### 2.4. Sucrose Preference Test (SPT)

The SPT method has been employed to evaluate the responses to stress that lead to a lack of pleasure [32]. In a cage, two bottles containing sterile drinking water were introduced and left undisturbed for a full day. For the actual test, one of these water bottles was swapped with one containing a 1% (*w*/*v*) sucrose solution, and the initial weights of the bottles were noted. To avoid any bias due to bottle placement, the positions of the bottles were alternated every six hours. After a 24 h period, the final weights of the bottles were recorded. The percentage of sucrose preference was then determined by calculating the mass difference.

### 2.5. Forced Swimming Test (FST)

The FST was conducted according to the established protocol [29]. The mice were introduced into a clear acrylic cylinder, measuring 20 cm in diameter and 40 cm in height. The cylinder was filled with water to a level of 19 cm and allowed to remain undisturbed for 6.5 min. The water was kept at a temperature of 22 °C throughout the procedure. The total duration of immobility during the final 5 min was then calculated for analysis.

### 2.6. Open Field Test (OFT)

The open field had sides measuring 50 cm each, with the area divided into a central and peripheral grid. The mouse was initially positioned at the field’s center. The duration the mouse remained in the central area over a 5 min period was noted to assess potential depressive-like behaviors in the mouse [33].

### 2.7. Tail Suspension Test (TST)

The mouse’s tail was secured with adhesive tape, and its behavior was captured using the Noldus EthoVision XT16 system for a duration of 6 min. The immobility period lasting the last 4 min was quantified [29].

### 2.8. Cell Culture and Treatment

The BV2 mouse microglia cell line was sourced from the American Type Culture Collection (ATCC). The cells were grown in Dulbecco’s Modified Eagle Medium (DMEM, Gibco, Grand Island, NY, USA) enriched with 10% fetal bovine serum (FBS, 10099141C, Gibco) and penicillin–streptomycin (100 U/mL, Biosharp, Beijing, China). They were kept at 37 °C in a humidified incubator with 5% CO_2_. The cells were exposed to 50 µM corticosterone (MedChemExpress, Monmouth Junction, NJ, USA) for 24 h.

### 2.9. Primary Culture of Microglia

Cells were isolated from the cerebral cortex, placed in 75 cm^2^ flasks, and cultured in Minimum Essential Medium (DMEM/F-12, 11320033, Gibco) with 10% FBS (10099141C, Gibco) and 1% penicillin–streptomycin (15070063, Gibco) at 37 °C. After 12–14 days of cultivation, mature microglia were detached from the mixed glial cultures by gentle shaking, resuspended in F12 with 10% FBS, and seeded into plates. The cells were allowed to settle for 2–3 h before further experimentation. For treatment, microglia were incubated with corticosterone [34].

### 2.10. 2-DG Treatment

2-DG (MedChemExpress, Monmouth Junction, NJ, USA), acting as a glycolytic inhibitor, was utilized to explore the possible role of heightened glycolytic activity in cognitive decline caused by CUMS. Mice received intraperitoneal injections of 2-DG (250 mg/kg, diluted in ddH_2_O) every two days during the final phase of the CUMS protocol (weeks 5–8) [35]. The control group was injected with an equivalent volume of ddH_2_O.

### 2.11. Enzyme-Linked Immunosorbent Assay (ELISA)

Hippocampal tissues were extracted from both stressed and control mice, mixed with prechilled PBS, and homogenized using a grinder. After centrifugation at 1000× *g* for 10 min at 4 °C, the supernatant was analyzed using a Mouse Corticosterone ELISA kit and NA/NE ELISA kit (Sangon, Shanghai, China). Hippocampal tissue supernatants were also evaluated using mouse InL-6, IL-1β, and TNF-α ELISA kits (ABclonal, Wuhan, China). The supernatant from cultured BV2 cells and primary microglia, after centrifugation at 1000 g for 15 min at 4 °C, was analyzed using mouse IL-6, IL-1β, and TNF-α ELISA kits (ABclonal, Wuhan, China) in accordance with the manufacturer’s guidelines [18].

### 2.12. Glucose Measurement of Hippocampus

Samples of hippocampal tissue were harvested and weighed from the brains of both stressed and control mice. Subsequently, 1 mL of distilled water was added per 0.1 g of tissue, and the mixture was homogenized using an electric homogenizer. The homogenate was incubated at 95 °C for 10 min and then centrifuged at 8000× *g* for 10 min. The supernatant was collected and its glucose content was determined using a Glucose Content Assay kit (Sangon Biotech, Shanghai, China), following the manufacturer’s protocol [18].

### 2.13. Immunofluorescence (IF) Staining

For immunohistofluorescence, brains were extracted and further fixed in 4% paraformaldehyde (PFA) at 4 °C overnight. After dehydration in a sucrose gradient from 10% to 30% (*w*/*v*) for cryoprotection, the brains were embedded in Tissue-Tek O.C.T. compound (#4583, SAKURA, Torrance, CA, USA). Sections of 25-µm thickness were cut using a Leica CM1950 (Leica Biosystems, Deer Park, IL, USA) cryostat and processed for immunohistofluorescence, as per established protocols [36].

For immunocytofluorescence, BV2 cells were treated as indicated for 24 h and then subjected to IF staining as previously reported [37]. Cells were fixed with 4% PFA and permeabilized with 0.2% Triton X-100. After three PBS washes, cells were blocked with 5% bovine serum albumin (BSA) in PBS for 1 h. Primary antibodies were applied in 2% BSA-containing PBS, followed by fluorescent secondary antibodies for detection. The antibodies used were IBA1 (1:200, Abcam, Cambridge, MA, USA, ab283319) and IL-1β (1:200, Abclonal, Wuhan, China, A16288). Secondary antibodies included goat anti-mouse IgG H&L (Alexa Fluor^®^ 488) (1:200, Abcam, Cambridge, MA, USA, ab150113) and goat anti-rabbit IgG H&L (Alexa Fluor^®^ 594) (1:200, Abcam, Cambridge, MA, USA, ab150080). Nuclei were counterstained with DAPI (Sigma Aldrich, St. Louis, MO, USA). Images were captured on a confocal laser scanning microscope (Olympus, Tokyo, Japan).

### 2.14. Quantitative Real-Time PCR (qRT-PCR)

Total RNA from BV2 cells, primary microglia, and hippocampal samples of mice was isolated using TRIzol reagent (#93289, Sigma-Aldrich) and reverse transcribed into cDNA with RT Master Mix (#G490, Abmart, Richmond, BC, Canada). qRT-PCR was performed on a LightCycler 96 Realtime PCR System (Roche, Basel, Switzerland) with TB Green Pre-mix Ex Taq kit (TaKaRa, Kyoto, Japan). β-Actin served as an endogenous control. Relative gene expression was calculated using the 2-ΔΔCt method [37]. Primer sequences are detailed in Supplementary Table S1.

### 2.15. Flow Cytometry Analysis

Intracellular ROS levels were determined using methods outlined previously [38]. After treatment, ROS levels were measured with 20,70-dichlorofluoresceindiacetate (H2DCF-DA, Thermo Fisher Scientific, Waltham, MA, USA). Cells were incubated with 20 μM DCFH-DA for 30 min at room temperature in the dark, washed with PBS, and analyzed for ROS production using a NovoCyte Flow Cytometer (Agilent Technologies, Santa Clara, CA, USA).

### 2.16. HK Activity Measurement

Activities of HK and PKM in hippocampal lysates were evaluated using assay kits from Abbkine (Atlanta, GA, USA), following the manufacturer’s guidelines.

### 2.17. Western Blotting

Hippocampus tissues or cells were lysed in RIPA buffer with a protease inhibitor cocktail (MedChemExpress, Monmouth Junction, NJ, USA) and centrifuged at 12,000× *g* for 20 min at 4 °C. Protein extracts (25 μg) were loaded onto 8–12% SDS-PAGE gels. Resolved proteins were transferred to PVDF membranes, blocked with 5% non-fat dry milk in TBST for 1 h, and incubated with primary antibodies against HK2 (1:1000, Cat# ab209847, Abcam), PKM2 (1:1000, Cat# 4053, Cell signaling technology, Danvers, MA, USA), and β-actin (1:1000, 66009-1-Ig, ProteinTech, Rosemont, IL, USA) overnight at 4 °C. Membranes were then incubated with HRP-conjugated secondary antibody (1:5000, R&D Systems, Abingdon, UK) for 1 h. An ECL Western blotting substrate Kit (Thermo Scientific, Waltham, MA, USA) was used for detection, followed by imaging on Image QuantLas 4000 (GE, Boston, MA, USA) and analysis with Image J software, RRID:SCR_003070, NIH, USA.

### 2.18. Metabolic Flux Analysis

The XF-24 Seahorse Extracellular Flux analyzer (Seahorse Bioscience, North Billerica, MA, USA) was used to monitor real-time ECAR and OCR changes in BV2 cells, as previously described [39]. Microglia were plated in XF-24 plates with or without corticosterone for 24 h, rinsed, and analyzed in XF running buffer for OCR or ECAR. Cell counts were determined post-measurement using DAPI staining (intelligent cell-normalized analysis system, Falcon S300, Alicelligent, Beijing, China). ATP production rates were estimated with the Agilent Seahorse XF ATP real-time rate assay [40]. Glycolytic ATP rates were calculated from lactate efflux using the proton prediction rate (PPR) provided by the XF24 Seahorse, corrected for respiratory CO_2_ acidification and geometric assay volume.

### 2.19. Statistical Analysis

Data analysis was conducted using GraphPad Prism 8.0, with the results expressed as mean ± SD. The normality of continuous variables was assessed. A two-tailed unpaired Student’s *t*-test was applied for comparisons between two independent groups, while one-way ANOVA with Tukey’s post hoc test was used for more than three groups. A *p*-value of less than 0.05 was considered statistically significant.

## 3. Results

### 3.1. Chronic Stress Induces Depressive-like Behavior in Mice and Leads to the Activation of Neuroinflammation

**Figure 1:**
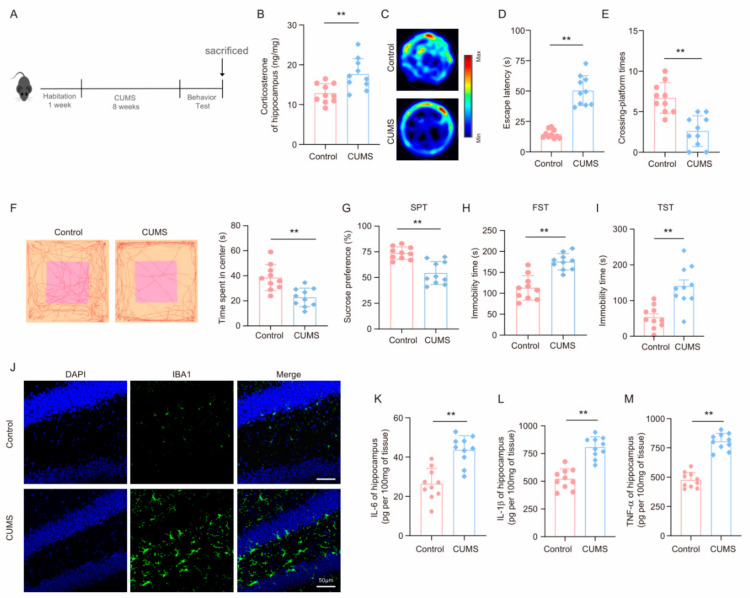
Chronic stress induces depressive-like behavior in mice and leads to the activation of neuroinflammation. (**A**) Schematic timeline of CUMS and behavior test. (**B**) Concentrations (ng/mg) of corticosterone in the hippocampus of control and stressed mice at the end of the CUMS procedure (n = 10, Student’s *t*-test, ** *p* < 0.01). (**C**) Representative track images of mice in the probe trial of MWM. (**D**,**E**) Escaping latency and crossing-platform times of mice (n = 10, Student’s *t*-test, ** *p* < 0.01). (**F**) Representative track images of mice in the Open Field Test (OFT) and time spent in the central (n = 10, Student’s *t*-test, ** *p* < 0.01). (**G**–**I**) Chronic unpredictable mild stress (CUMS)-induced depression-like behaviors were assessed by sucrose preference in the sucrose preference test (SPT) (**G**), immobility time in the forced swimming test (FST) (**H**), and immobility time in the Tail Suspension Test (TST) (**I**) (n = 10, Student’s *t*-test, ** *p* < 0.01). (**J**) Representative images of IF staining of hippocampal sections from control and CUMS mice. Iba-1, green; DAPI, blue. Scale bar, 50 μm. (**K**–**M**) Levels of IL-6, IL-1β, and TNF-α in hippocampus lysates from control and CUMS mice as determined by ELISA (n = 10, Student’s *t*-test, ** *p* < 0.01).

Upon completion of the 8-week CUMS program (Figure 1A), assessments of key hormones and cognitive functions were conducted to evaluate the effects of chronic stress on brain health. As depicted in Figure 1B and Figure S1A, stressed mice exhibited elevated corticosterone levels in the hippocampus compared to controls, while norepinephrine levels remained unchanged, aligning with the notion that glucocorticoids predominantly drive the damage caused by CUMS [41]. Most notably, in the MWM experiment, CUMS-exposed mice required more time to locate the hidden platform than their non-stressed counterparts (Figure 1C,D). Furthermore, during the probe trial, CUMS mice had fewer platform crossings (Figure 1E). In the OFT, these mice also spent less time in the central grid than control mice (Figure 1F). Additionally, CUMS mice displayed reduced sucrose preference and increased immobility in the FST and TST (Figure 1G–I). Collectively, these findings suggest that chronic stress can lead to depressive-like behaviors in mice.

The hippocampus is crucial for depressive-like behaviors. We explored how chronic stress affects microglia in this region. At the end of the CUMS program, hippocampal tissues were collected. Immunofluorescence analysis using the microglia marker Iba-1 revealed that CUMS enhanced microglia activation (Figure 1J). We also assessed the influence of chronic stress on hippocampal inflammatory factors. mRNA levels of the pro-inflammatory cytokines IL-6, IL-1β, and TNF-α were higher in CUMS mice (Figure S1B–D). Moreover, ELISA confirmed significant upregulation of these inflammatory markers (IL-6, IL-1β, and TNF-α) in the hippocampus of CUMS mice (Figure 1K–M). In conclusion, these data indicate that chronic stress not only triggers depressive-like behaviors in mice but also triggers neuroinflammation.

### 3.2. Chronic Stress Induces Upregulation of Key Enzymes of Glycolysis in the Mouse Hippocampus

**Figure 2:**
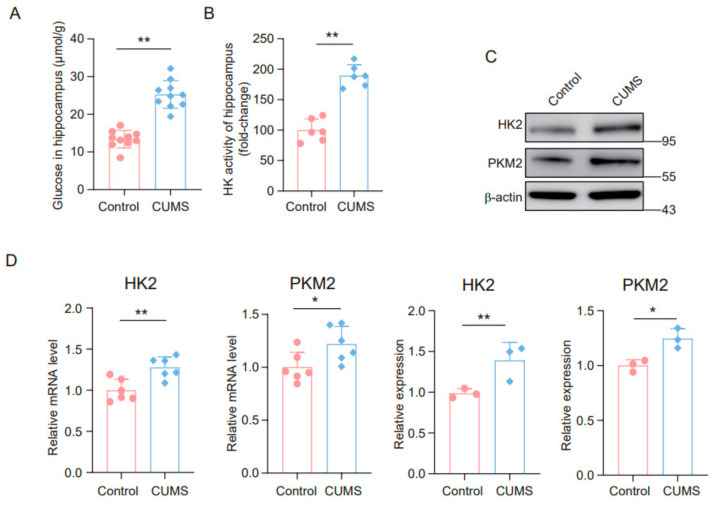
Chronic stress induces the upregulation of key enzymes of glycolysis in the mouse hippocampus. (**A**) Glucose levels in hippocampus lysates from control and CUMS mice (n = 10, Student’s *t*-test, ** *p* < 0.01). (**B**) HK activity in hippocampus lysates from control and CUMS mice (n = 6, Student’s *t*-test, ** *p* < 0.01). (**C**) Western blot of the expression of HK2 and PKM2 protein in hippocampus lysates from control and CUMS mice (n = 3, Student’s *t*-test, * *p* < 0.05, ** *p* < 0.01). (**D**) qRT-PCR assays monitoring the expression of HK2 and PKM2 in hippocampal lysates from control and CUMS mice (n = 6, Student’s *t*-test, * *p* < 0.05, ** *p* < 0.01).

First, we explored whether hippocampal glycolysis in CUMS mice was altered by measuring hippocampal glucose levels. In line with prior studies [18], we observed a significant increase in glucose levels within the hippocampal tissue of CUMS mice (Figure 2A). Moreover, we evaluated the activity of HK, a crucial enzyme in glycolysis, in hippocampal tissue extracts from the mice. The findings indicated that HK activity in the hippocampus of CUMS mice was markedly higher than in control mice (Figure 2B). We further analyzed the protein levels of key glycolytic enzymes and discovered that the levels of HK2 and PKM2 were increased in the CUMS model (Figure 2C). Additionally, real-time quantitative PCR demonstrated that mRNA levels of HK2 and PKM2 were higher in the hippocampi of CUMS mice compared to the control group (Figure 2D). Collectively, these results suggest that the CUMS animal model exhibits augmented glycolytic activity in the hippocampus.

### 3.3. Exposure to High Concentrations of Corticosterone Promotes the Inflammatory Response of Microglia

**Figure 3:**
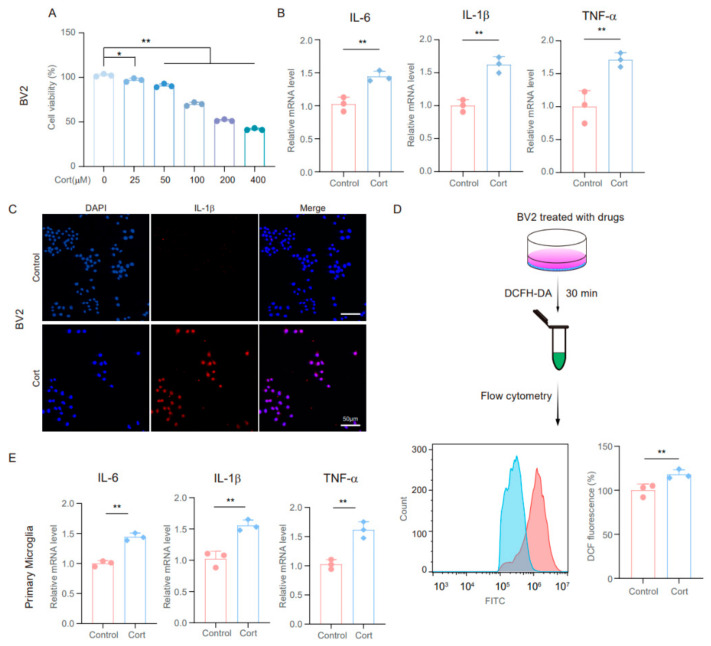
Exposure to high concentrations of corticosterone promotes the inflammatory response of microglia. (**A**) BV2 cells were treated with the indicated concentrations of Cort for 24 h, and cell growth was evaluated using a cell counting kit-8 (CCK-8) assay in three independent experiments; one-way ANOVA with Tukey’s post hoc test, * *p* < 0.05, ** *p* < 0.01. (**B**) qRT-PCR assays monitoring the expression of inflammatory factors, IL-1β, IL-6, and TNF-α in BV2 cells incubated with Cort (50 µmol/L) for 24 h. Control cells were incubated with DMSO (n = 3, Student’s *t*-test, ** *p* < 0.01). (**C**) Representative images of IL-β staining in Cort-treated BV2 cells and control cells. Scale bar, 50 μm. (**D**) BV2 cells were treated with Cort for 24 h. Intracellular ROS levels were quantified using DCFH-DA (n = 3, Student’s *t*-test, ** *p* < 0.01). (**E**) qRT-PCR assays monitor the expression of inflammatory factors, IL-1β, IL-6, and TNF-α in primary microglia cells incubated with Cort (50 µmol/L) for 24 h. Control cells were incubated with DMSO (n = 3, Student’s *t*-test, ** *p* < 0.01).

It is widely accepted that glucocorticoids (GCs) play a central role in the damage caused by chronic stress. To assess the impact of corticosterone on cell viability, we exposed BV2 cells to varying concentrations of corticosterone for 24 h. Figure 3A illustrates that cell viability was reduced in a concentration-dependent manner following treatment. To further explore the influence of corticosterone on neuroinflammatory responses, BV2 and primary microglia cells were treated with corticosterone at a concentration of 50 μM. The findings revealed that corticosterone markedly upregulated the expression of intracellular inflammatory factors such as IL-6, IL-1β, and TNF-α (Figure 3B,E). Additionally, ELISA confirmed significant increases in the levels of these inflammatory factors in BV2 and primary microglia cells exposed to corticosterone (Figure S2A–F). Immunofluorescence staining also showed higher intracellular IL-1β levels after corticosterone treatment (Figure 3C). Furthermore, since activated microglia are known to generate intracellular ROS [42], we quantified intracellular ROS levels using DCFH-DA. A significant increase in ROS levels was observed in BV2 cells following corticosterone stimulation (Figure 3D). In summary, these results demonstrate that corticosterone triggers a pro-inflammatory response in BV2 and primary microglia cells.

### 3.4. Exposure to High Corticosterone Promotes Increased Glycolysis in Microglia

**Figure 4:**
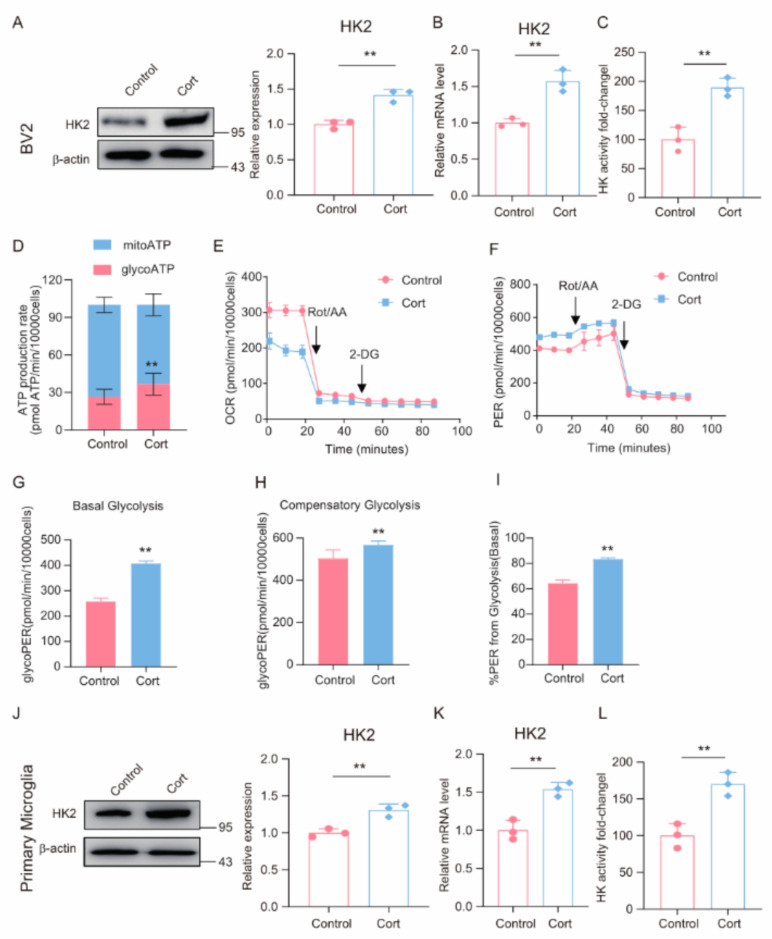
Exposure to high corticosterone promotes increased glycolysis in microglia. (**A**–**J**) Western blot of the expression of HK2 protein in BV2 and primary microglia cells lysates from control and Cort. Control cells were incubated with DMSO (n = 3, Student’s *t*-test, ** *p* < 0.01). (**B**,**K**) qRT-PCR assays monitoring the expression of HK2 level in BV2 and primary microglia cells incubated with Cort (50 µmol/L) for 24 h. (n = 3, Student’s *t*-test, ** *p* < 0.01). (**C**,**L**) HK activity in BV2 and primary microglia cells lysates from control and Cort (n = 3, Student’s *t*-test, ** *p* < 0.01). (**D**) Seahorse Extracellular Flux analysis to quantify ATP production in BV2 cells (n = 3, Student’s *t*-test, ** *p* < 0.01). (**E**–**I**) Treatment with Cort for 24 h significantly inhibited OCR in BV2 cells. Treatment with Cort for 24 h significantly increased glycolytic capacity of BV2 cells. Seahorse Extracellular Flux analysis to quantify basal and maximal respiration in BV2 cells. All the experiments were performed by the Agilent’s Seahorse Bioscience XF24 Extracellular Flux Analyzer. (n = 3, Student’s *t*-test, ** *p* < 0.01).

Glucocorticoids, renowned for their pivotal role in stress responses, are integral in modulating glucose metabolism by curbing glucose uptake and enhancing its breakdown. Considering the significant function of aerobic glycolysis in the regulation of inflammatory cells [43], we sought to determine if corticosterone could modulate this metabolic pathway in microglia cells. We observed an increase in the protein and mRNA levels of the principal glycolytic enzyme HK2 in BV2 and primary microglia cells following corticosterone exposure (Figure 4A,B,J–K). Additionally, we noted a rise in HK activity in these corticosterone-treated cells (Figure 4C,L). To further assess the metabolic effects, we employed the Seahorse assay to quantify ATP generation, along with the measurement of baseline OCR, basal respiration, and maximal respiration in corticosterone-treated BV2 cells. The findings indicated a surge in glycolytic ATP production in comparison to mitochondrial ATP production in BV2 cells post-corticosterone treatment (Figure 4D). Moreover, the OCR of BV2 cells was diminished compared to the control, while the proton efflux rate, basal respiration, and maximal respiration were all observed to increase. In conclusion, these results imply that corticosterone can stimulate an escalation in glycolytic activity within BV2 cells (Figure 4E–I).

### 3.5. 2-DG Ameliorates Corticosterone-Induced Microglia Inflammation by Inhibiting Glycolysis

**Figure 5:**
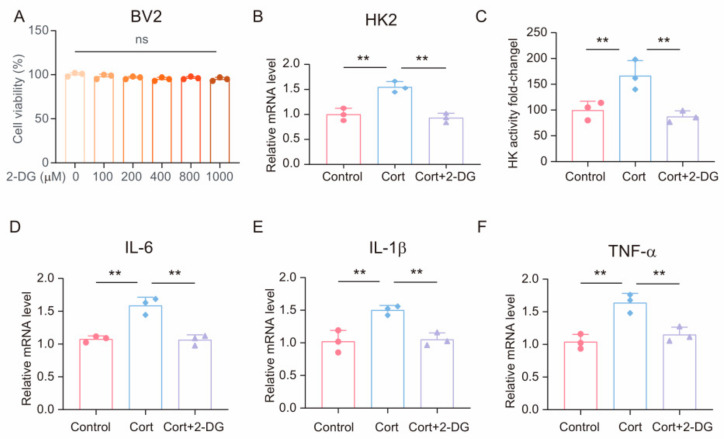
2-DG ameliorates corticosterone-induced microglia inflammation by inhibiting glycolysis. (**A**) BV2 cells were treated with the indicated concentrations of 2-DG for 24 h, and cell growth was evaluated using a cell counting kit-8 (CCK-8) assay in three independent experiments; one-way ANOVA with Tukey’s post hoc test, ns: *p* > 0.05. (**B**) qRT-PCR assays monitoring the expression of HK2 level in BV2 cells incubated with Cort (50 µmol/L) or 2-DG (1 mmol/L) for 24 h. (n = 3, one-way ANOVA with Tukey’s post hoc test, ** *p* < 0.01). (**C**) HK activity in BV2 cells lysates from control, Cort, and Cort +2-DG (n = 3, one-way ANOVA with Tukey’s post hoc test, ** *p* < 0.01). (**D**–**F**) qRT-PCR assays monitoring the expression of inflammatory factors, IL-6, IL-1β, and TNF-α in BV2 cells incubated with Cort (50 µmol/L) or 2-DG (1 mmol/L) for 24 h. Control cells were incubated with DMSO (n = 3, one-way ANOVA with Tukey’s post hoc test, ** *p* < 0.01).

We subjected BV2 cells to various concentrations of 2-DG for a 24 h period to ascertain its impact on cell viability [44]. As depicted in Figure 5A, treatment with 2-DG did not exert any detrimental effects on microglia viability. To further explore the influence of 2-DG on the production of pro-inflammatory cytokines, BV2 cells were cotreated with 2-DG (1000 μM) and corticosterone (50 μM). Initially, we observed that the mRNA levels of HK2 and the enzymatic activity of HK were reduced in the group treated with 2-DG compared to those treated solely with corticosterone (Figure 5B,C). Moreover, real-time quantitative PCR data revealed a significant downregulation of mRNA expression for inflammatory markers, including IL-6, IL-1β, and TNF-α, in cells subjected to 2-DG treatment (Figure 5D–F). This observation suggests that the suppression of glycolysis can mitigate the inflammatory response of microglia.

### 3.6. 2-DG Ameliorates CUMS-Induced Neuroinflammation and Depressive-Like Behavior in Mice by Inhibiting Glycolysis

**Figure 6:**
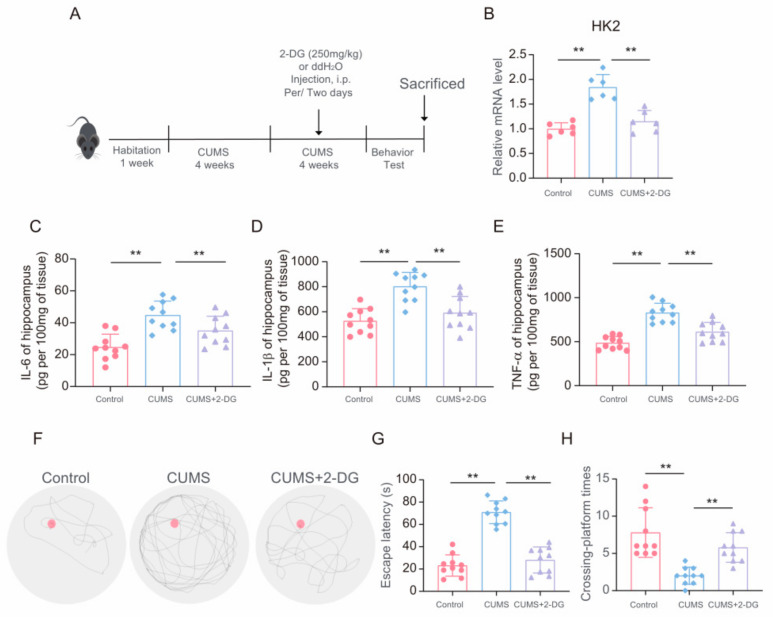
2-DG ameliorates chronic stress-induced neuroinflammation and depressive-like behavior in mice by inhibiting glycolysis. (**A**) Schematic timeline of CUMS, 2-DG treatment, and behavior test. (**B**) qRT-PCR assays monitoring the expression of HK2 in hippocampal lysates from control and CUMS mice (n = 6, one-way ANOVA with Tukey’s post hoc test, ** *p* < 0.01). (**C**–**E**) Levels of IL-1β, IL-6 and TNF-α in hippocampus lysates from control, CUMS, and CUMS+2-DG mice as determined by ELISA (n = 10, one-way ANOVA with Tukey’s post hoc test, ** *p* < 0.01). (**F**–**H**) Representative track images of mice in the probe trial of MWM, escaping latency and crossing-platform time of mice (n = 10, one-way ANOVA with Tukey’s post hoc test, ** *p* < 0.01).

To delve deeper into the impact of 2-DG on depressive-like symptoms and neuroinflammation stemming from chronic stress, we administered 2-DG to CUMS mice to curb their glycolytic activity (Figure 6A). The results showed that 2-DG could alleviate the increase in glycolysis induced by CUMS in the hippocampus of mice (Figure 6B). Subsequently, we utilized ELISA to measure the levels of inflammatory cytokines (IL-6, IL-1β, and TNF-α) in the hippocampal tissue extracts of the mice, discovering that 2-DG could lessen the neuroinflammation provoked by chronic stress (Figure 6C–E). Additionally, the mRNA levels of inflammatory mediators in the hippocampus were also found to be reduced by 2-DG treatment (Figure S3A–C). Although differences still existed between stress-treated mice and control mice under 2-DG treatment, the MWM task results showed that 2-DG could ameliorate the escape latency elongated by CUMS (Figure 6F,G), and the platform crossing count in CUMS mice with 2-DG treatment was notably higher (Figure 6H). Furthermore, in the Open Field Test, CUMS mice treated with 2-DG spent more time in the central zone (Figure S3D,E). Collectively, these outcomes propose that 2-DG can mitigate both neuroinflammation and depressive-like behaviors induced by chronic stress.

## 4. Discussion

This study provides evidence that 8 weeks of chronic stress lead to elevated aerobic glycolysis and neuroinflammation in the hippocampus of mice. As a glycolysis inhibitor, 2-DG can regulate glycolysis levels and the production of inflammatory cytokines. In vitro, we found that the stress hormone corticosterone can induce inflammatory responses in BV2 and primary microglia cells. 2-DG impairs the synthesis of corticosterone-induced pro-inflammatory mediators by inhibiting aerobic glycolysis. Our study reveals that 2-DG can alleviate the heightened glycolysis in the hippocampus caused by chronic stress. Additionally, we showed that systemic administration of 2-DG effectively suppresses neuroinflammation and alleviates depressive-like behaviors triggered by chronic stress.

Chronic stress is a prolonged state of psychological or physiological pressure that differs from transient acute stress due to its persistence, potentially lasting for weeks, months, or even years. Chronic stress is typically caused by long-term psychosocial factors such as work pressure, family issues, financial difficulties, prolonged illness, or social isolation. This stress state has extensive and profound impacts on human health, involving complex responses from multiple physiological systems [45]. Chronic stress is considered a significant factor in triggering neuroinflammation. A multitude of clinical and preclinical investigations have established a robust link between neuroinflammation and the depressive-like behaviors that result from chronic stress [46]. The CUMS paradigm is frequently employed to model depressive-like traits in animals. Nevertheless, the etiology of depression associated with stress is not yet completely comprehended [47,48,49]. Some studies suggest that targeting neuroinflammation can alleviate cognitive dysfunction [50,51]. Our findings are consistent with many studies, showing that chronic stress can activate microglia cells in the hippocampus and induce their polarization to a pro-inflammatory phenotype. Additionally, our results indicate that chronic stress can induce depressive-like phenotypes in mice.

The dysregulation of glucose metabolism and neuroinflammation comprises two major contributors to depression. Clinical studies indicate that the prevalence of depression among diabetic patients is twice that of the general population, and this condition triggers a robust inflammatory response in retinal and myocardial cells [52,53]. Glucocorticoids, the primary stress hormones, are well-known for their role in regulating glucose metabolism by inhibiting glucose consumption, promoting gluconeogenesis, and inducing hyperglycemia. Previous research from our lab, along with other studies, suggests that changes in glucose metabolism are involved in regulating hippocampal neuroinflammation [18,54]. In CUMS-induced hippocampal hyperglycemia, GLUT1-mediated activation of microglia pro-inflammatory factors promotes neuroinflammation [18]. There are clear connections and complex interactions between inflammation, glucose metabolism, and chronic stress. Systemic low-grade inflammation is a common characteristic of chronic metabolic disorders and stress-related diseases [55]. Evidence indicates that injecting TNF-α into the hippocampus can lead to depressive-like behaviors, while IL-1β also promotes depression onset, highlighting the role of central inflammatory factors in depression [30,56]. Additionally, metabolic disorders have been shown to drive neuroinflammation, contributing to the development of neurodegenerative diseases [57]. During immune challenges, cells tend to rely on glycolysis rather than mitochondrial pathways to conserve and produce metabolic resources, leading to increased lactate production, glucose uptake, activation of the pentose phosphate pathway (PPP), and reduced mitochondrial oxygen consumption [58]. In this study, our data reveal that chronic stress elevates glycolysis levels in the hippocampus of mice. Moreover, the stress hormone corticosterone induces inflammatory responses in BV2 and primary microglia cells. Consistent with our findings, other research has demonstrated that hyperglycemia promotes the pro-inflammatory polarization of hepatic macrophages or brain microglia, exacerbating inflammation in both the liver and brain [59,60]. We also found that corticosterone significantly increases the ECAR in BV2 cells and boosts glycolytic ATP production.

In the presence of oxygen, glucose can be converted to lactate through fermentation without entering the tricarboxylic acid cycle, a process called aerobic glycolysis. This phenomenon is also observed in some tumor cells. 2-DG treatment induces apoptosis in tumor cells that rely on glycolysis, whereas aerobic cells with functional mitochondria can survive glycolysis inhibition [61]. In our study, 2-DG treatment reduced neuroinflammation caused by chronic stress. During immune activation, cellular metabolism shifts from oxidative phosphorylation (OXPHOS) to glycolysis, even though glycolysis produces significantly less ATP than OXPHOS [62]. Resting microglia monitor their environment for potential threats, and when exposed to harmful conditions, they become activated to restore homeostasis. Resting microglia primarily depend on OXPHOS for energy but switch to glycolysis upon activation [63]. Research has shown that exposure to amyloid-β causes microglia to reprogram their metabolism from OXPHOS to glycolysis. Glycolysis is also upregulated in LPS-activated microglia, along with increased inflammatory factor release. Following 2-DG treatment, the inhibition of glycolysis suppresses microglia activation and reduces inflammatory factor expression [13,64]. Our findings suggest that 2-DG can mitigate microglia inflammation and alleviate depressive-like behaviors induced by CUMS by inhibiting glycolysis.

## 5. Conclusions

In conclusion, the findings from this study show that an 8-week CUMS protocol increases glycolysis in the hippocampus of mice and triggers microglial inflammatory responses. The glycolysis inhibitor 2-DG reduces neuroinflammation and depressive-like behaviors by regulating glycolysis. These results establish a connection between glycolysis and neuroinflammation caused by chronic stress, and further investigation of these mechanisms will provide deeper insight into the role of glycolysis in chronic stress.

## Data Availability

The datasets generated and/or analyzed during the current study are available from the corresponding author on reasonable request. The data are not publicly available due to specific ethical and privacy considerations.

## Supplementary Materials

The following supporting information can be downloaded at: https://www.mdpi.com/article/10.3390/brainsci14111098/s1, Figure S1: Chronic stress induces depressive-like behavior in mice and leads to the activation of neuroinflammation. Figure S2: High corticosterone exposure promotes inflammatory response in the microglia. Figure S3: 2-DG ameliorates chronic stress-induced neuroinflammation and depressive-like behavior in mice by inhibiting glycolysis. Table S1: Sequences of qPCR primers.

## Abbreviations

2-deoxy-D-glucose (2-DG); Corticosterone (Cort); Reactive Oxygen Species (ROS); Hexokinase 2 (HK2); Central Nervous System (CNS); Hypothalamic–Pituitary–Adrenal (HPA); Interleukin-6 (IL-6); Interleukin-1β (IL-1β); Tumor Necrosis Factor-α (TNF-α); Lipopolysaccharide (LPS); Oxygen Consumption Rate (OCR); Morris Water Maze (MWM); Hexokinase (HK); Sucrose Preference Test (SPT); Forced Swimming Test (FST); Open Field Test (OFT); Tail Suspension Test (TST); American Type Culture Collection (ATCC); Dulbecco’s Modified Eagle Medium (DMEM); Fetal Bovine Serum (FBS); Enzyme-linked Immunosorbent Assay (ELISA); Immunofluorescence (IF); Paraformaldehyde (PFA); Bovine Serum Albumin (BSA); Quantitative Real-Time PCR (qRT-PCR); Proton Prediction Rate (PPR); Glucocorticoids (GC); Pentose Phosphate Pathway (PPP); Oxidative Phosphorylation (OXPHOS)
