# Supplementary material for: Inhibition of Glycolysis Alleviates Chronic Unpredictable Mild Stress Induced Neuroinflammation and Depression-like Behavior
Source: Brain Sci. 2024 Oct 30;14(11):1098. doi: 10.3390/brainsci14111098 (PMC11591872; doi:10.3390/brainsci14111098)
Supplement: Supplementary file 1 [file brainsci-14-01098-s001.zip › brainsci-3261386-supplementary.pdf]

Figure legend

Figure S1. Chronic stress induces depressive-like behavior in mice and leads to the activation of neuroinflammation.

(A) Concentrations (ng/mg) of norepinephrine in the hippocampus tissues of Control and stressed mice at the end of the CUMS procedure (n = 10, Student's t-test).

(B-D) qRT-PCR assays monitoring the expression of inflammatory factors, IL-6, IL-1β and TNF-α in hippocampus lysates from Control and CUMS mice. (n = 6, Student's t-test, * *$p < 0.01$).

Figure S2. High corticosterone exposure promotes inflammatory response in the microglia.

(A-C) Levels of IL-1β, IL-6 and TNF-α in BV2 cells from Control and Cort as determined by ELISA (n = 3, Student's t-test, *$p < 0.05$).

(D-E) Levels of IL-1β, IL-6 and TNF-α in Primary Microglia cells from Control and Cort as determined by ELISA (n = 3, Student's t-test, *$p < 0.05$).

Figure S3. 2-DG ameliorates chronic stress-induced neuroinflammation and depressive-like behavior in mice by inhibiting glycolysis.

(A-C) qRT-PCR assays monitoring the expression of inflammatory factors, IL-1β, IL-6 and TNF-α in hippocampus lysates from Control, CUMS and CUMS+2-DG mice (n = 6, One-way ANOVA with Tukey's post hoc test, * *$p < 0.01$).

(D&E) Representative track images of mice in the open field and time spent in the central of OFT (n = 10, One-way ANOVA with Tukey's post hoc test, * *$p < 0.01$).

Supplementary Table S1 Sequences of qPCR primers

Gene name	Forward	Reverse
HK2	ATTGTGGCTGTGGTGAA	AATGTGACGCATCTCCTC
PKM2	AGGCTGCCATCTACCACTTG	CACTGCAGCACTTGAAGGAG
IL-1β	CAGGCAGGCAGTATCACTCATTG	GCTTTTTTGTTGTTCATCTCGGA
IL-6	TAGTCCTTCCTACCCCAATTTCC	TTGGTCCTTAGCCACTCCTTC
TNF-α	ACGGCATGGATCTCAAA	AGATAGCAAATCGGCTGAC
β-actin	GGCTGTATTCCCCTCCATCG	CCAGTTGGTAACAATGCCATGT

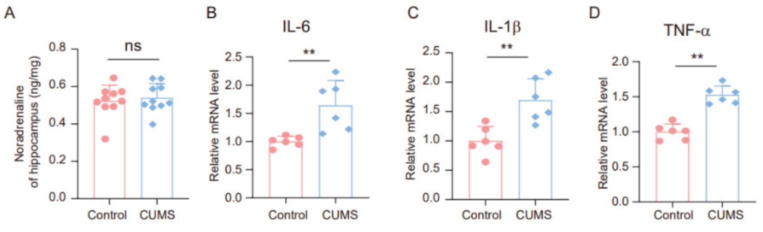

Figure S1

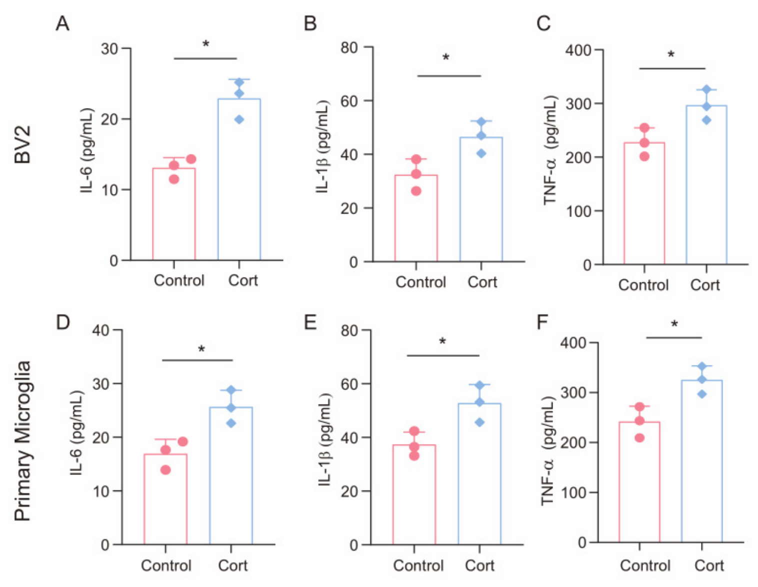

Figure S2

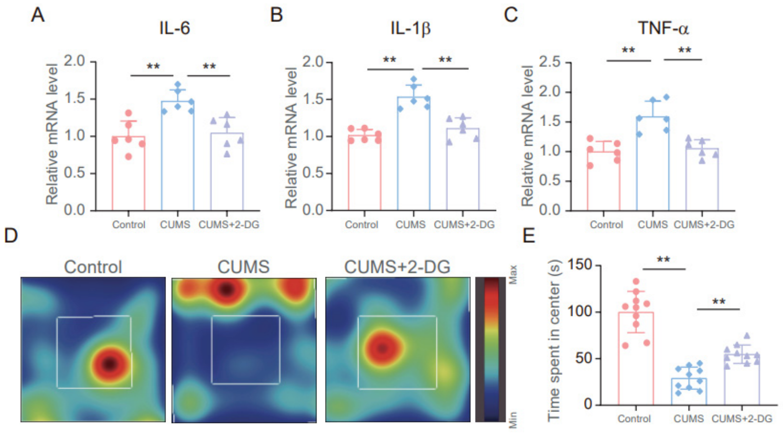
A
IL-6
B
IL-1β
C
TNF-α
Relative mRNA level
Control
CUMS
CUMS+2-DG
D
E
Max
Min
Time spent in center (s)
**

Figure S3
